# High-dose accelerated hypofractionated three-dimensional conformal radiotherapy (at 3 Gy/fraction) with concurrent vinorelbine and carboplatin chemotherapy in locally advanced non-small-cell lung cancer: a feasibility study

**DOI:** 10.1186/1748-717X-8-198

**Published:** 2013-08-11

**Authors:** Yue-E Liu, Qiang Lin, Fan-Jie Meng, Xue-Ji Chen, Xiao-Cang Ren, Bin Cao, Na Wang, Jie Zong, Yu Peng, Ya-Jun Ku, Yan Chen

**Affiliations:** 1 Department of Oncology, North China Petroleum Bureau General Hospital of Hebei Medical University, 8 Huizhan Avenue, Renqiu, Hebei Province 062552, P.R. China

**Keywords:** Non-small-cell lung cancer, Accelerated hypofractionated radiotherapy, Concurrent chemoradiotherapy, Three-dimensional conformal radiotherapy, Vinorelbine, Carboplatin

## Abstract

**Background:**

Increasing the radiotherapy dose can result in improved local control for non-small-cell lung cancer (NSCLC) and can thereby improve survival. Accelerated hypofractionated radiotherapy can expose tumors to a high dose of radiation in a short period of time, but the optimal treatment regimen remains unclear. The purpose of this study was to evaluate the feasibility of utilizing high-dose accelerated hypofractionated three-dimensional conformal radiotherapy (at 3 Gy/fraction) with concurrent vinorelbine (NVB) and carboplatin (CBP) chemotherapy for the treatment of local advanced NSCLC.

**Methods:**

Untreated patients with unresectable stage IIIA/IIIB NSCLC or patients with a recurrence of NSCLC received accelerated hypofractionated three-dimensional conformal radiotherapy. The total dose was greater than or equal to 60 Gy. The accelerated hypofractionated radiotherapy was conducted once daily at 3 Gy/fraction with 5 fractions per week, and the radiotherapy was completed in 5 weeks. In addition to radiotherapy, the patients also received at least 1 cycle of a concurrent two-drug chemotherapy regimen of NVB and CBP.

**Results:**

A total of 26 patients (19 previously untreated cases and 7 cases of recurrent disease) received 60Gy-75Gy radiotherapy with concurrent chemotherapy. All of the patients underwent evaluations for toxicity and preliminary therapeutic efficacy. There were no treatment-related deaths within the entire patient group. The major acute adverse reactions were radiation esophagitis (88.5%) and radiation pneumonitis (42.3%). The percentages of grade III acute radiation esophagitis and grade III radiation pneumonitis were 15.4% and 7.7%, respectively. Hematological toxicities were common and did not significantly affect the implementation of chemoradiotherapy after supportive treatment. Two patients received high dose of 75 Gy had grade III late esophageal toxicity, and none had grade IV and above. Grade III and above late lung toxicity did not occur.

**Conclusion:**

High-dose accelerated hypofractionated three-dimensional conformal radiotherapy with a dose of 60 Gy or greater with concurrent NVB and CBP chemotherapy might be feasible. However esophagus toxicity needs special attention. A phase I trial is recommended to obtain the maximum tolerated radiation dose of accelerated hypofractionated radiotherapy with concurrent chemotherapy.

## Background

Concurrent chemoradiotherapy is one of the main means of treatment for locally advanced non-small-cell lung cancer (NSCLC) [1] and has been proven to be superior to sequential chemotherapy and radiotherapy [2]. Currently, it is believed that concurrent chemoradiotherapy improves survival mainly by improving local control [3]. Therefore, increasing the radiation dose can further improve local control and subsequently improve survival.

Dose-escalation studies of conventional fractionated radiotherapy with concurrent chemotherapy demonstrated that, although phase I/II trials reported encouraging survival outcomes obtained with the high dose of 74 Gy [4,5], the results of the subsequent phase III randomized controlled study were disappointing and difficult to comprehend. The survival outcomes for the 74 Gy high-dose group were not improved or were even less favorable than the 60 Gy standard-dose group, with 1-year overall survival (OS) rates of 70.4% and 81%, respectively. The preliminary analysis revealed no significant differences in toxicity between the high-dose group and the low-dose group [6]; therefore, the above OS results cannot be explained by excessive treatment toxicity for the high-dose group. NSCLC is a rapidly proliferating cancer, and accelerated repopulation occurs during radiotherapy. If the treatment duration extends for longer than 6 weeks, each additional day of treatment is associated with a 1.6% decrease in survival [7]. Although the exact reasons why high-dose radiotherapy in the Radiation Therapy Oncology Group (RTOG) 0617 study failed to produce survival benefits remain unclear, one potential factor that should be considered is the long treatment time of 7.4 weeks for the conventional fractionation [1].

If the radiation dose for each fraction is increased, and the entire radiotherapy regimen is completed with 25 fractions and within the total treatment time of 5 weeks, this radiotherapy regimen could lead to a 10%-15% greater local control than that of conventional fractionated radiotherapy at 2 Gy/fraction without increasing late toxicity [7]. Because accelerated hypofractionation can significantly shorten the total treatment time and reduce the number of treatments, as well as provide the advantages of convenience and economy, the regimen of hypofractionated radiotherapy and concurrent chemotherapy should have good clinical prospects in theory. However, due to concerns of treatment-related toxicity, there have been a limited number of reports on hypofractionated radiotherapy with concurrent chemotherapy. With the advances in modern precision radiotherapy techniques and the widespread clinical application of these regimens [8], the three-dimensional conformal radiotherapy technique can be used to conduct hypofractionated radiotherapy, which can significantly lower the radiation doses that normal tissues receive and thereby make it possible to combine radiotherapy with concurrent chemotherapy [9-13].

For locally advanced NSCLC, a 3-Gy dose of accelerated hypofractionated radiation alone is often applied [14-17], and under certain conditions, the radiation dose can be increased to 75 Gy [17]. To our knowledge, there have been no reports regarding phase I/II studies for hypofractionated radiotherapy at 3 Gy/fraction with concurrent chemotherapy. The maximum dose to which the radiotherapy can be escalated is thus unclear. The purpose of our prospective small-sample study was to evaluate the feasibility of utilizing high-dose accelerated hypofractionated three-dimensional conformal radiotherapy (at 3 Gy/fraction) with concurrent vinorelbine (NVB) and carboplatin (CBP) chemotherapy for the treatment of locally advanced NSCLC.

## Methods

### Inclusion criteria

Patients either had been pathologically or cytologically confirmed to have previously untreated NSCLC and were at the clinical stage of unresectable stage IIIA or stage IIIB NSCLC [as defined by the 2009 staging standards of the International Union Against Cancer (UICC)] or had a recurrence after surgery. The patients were aged older than or equal to 18 years and no more than 75 years. The Karnofsky performance status (KPS) scores were at least 70, and the expected survival time was at least 3 months. The laboratory test results were as follows: neutrophil counts were 2.0×10^9^/L; hemoglobin levels were at least 100 g/L; platelet counts (PLTs) were at least 100×10^9^/L; and serum creatinine, alanine aminotransferase, aspartate aminotransferase, and total bilirubin levels were below the upper limit of the normal range. The patients exhibited no significant electrocardiographic (ECG) abnormalities, had no history of serious heart disease, could receive concurrent chemoradiotherapy, and did not require hospitalization for diseases other than NSCLC.

### Exclusion criteria

Patients were excluded from the study if they were pregnant or lactation or had other malignancies (with the exception of cases of cervical carcinoma in situ or non-malignant melanoma skin cancer that had been clinically cured for at least 5 years). Patients who could not undergo concurrent chemoradiotherapy for medical reasons or who suffered from either superior vena cava syndrome or severe lung disorders that affected lung function were also excluded.

This clinical trial was approved by the Ethics Committee of Hebei Medical University. This study was performed in accordance with the standards for human clinical trials and the principles stated in the Declaration of Helsinki (as issued in 1975 and revised in 2000). All of the patients signed an informed consent form prior to enrollment.

### Patient assessment

Patients underwent assessments within 2 weeks prior to the treatment, including a complete medical history, a comprehensive physical examination, head, thoracic and abdominal contrast-enhanced computed tomography (CT) or magnetic resonance imaging (MRI) of the head, an ECG, a bronchoscopy, routine blood testing, and a comprehensive blood biochemical profile. If clinically indicated, a whole-body bone scan was performed with emission CT (ECT).

The patients received physical examinations and had routine blood testing performed each week (with an increased frequency of examinations if necessary). Full biochemical profiles were obtained and an ECG was performed prior to each chemotherapy cycle.

### Study design

This study was a single-arm, prospective, non-randomized feasibility study. The primary research aim was to investigate whether accelerated hypofractionated three-dimensional conformal radiotherapy (at 3 Gy/fraction) with a total dose of 60 Gy or greater with concurrent NVB and CBP chemotherapy could be safely applied. The secondary aims included investigation of the short-term efficacy, the progression-free survival (PFS), and the OS. The chemoradiotherapy treatment scheme is depicted in Table 1.

**Table 1:** Concurrent chemoradiotherapy schema

**Concurrent chemoradiotherapy schema**						
RT regimen: Weeks 1–5: 3 Gy/f, 1 f/d,5 f/w;					Dose level	
Week	1	2	3	4	5	6
RT	**┃┃┃┃┃**	**┃┃┃┃┃**	**┃┃┃┃┃**	**┃┃┃┃┃**	**60 Gy:none**	
					**63 Gy:┃**	
					**69 Gy: ┃┃┃**	
					**75 Gy: ┃┃┃┃┃**	
Chemotherapy: NVB (25 mg/m2) d1, d8; CBP, AUC = 5 mg/m1.min on d8, repeated every 28 d						
NVB	◆	◆			◆	◆
CBP		●				●

### Radiation therapy

The patients were in the supine position with their hands folded on top of their heads. A vacuum pad was used to immobilize the patient’s body position and appropriately limit respiratory motion. Contrast-enhanced spiral CT (Volume Computed Tomography, GE Healthcare, Milwaukee, WI, USA) was performed in the same body position as the treatment. The image data were input into the three-dimensional treatment planning system. The Venus 5014 software package (Shanghai Tuoneng Co., Shanghai, China) was used to design the radiotherapy plan, which utilized convolution algorithms. The delineation of the thoracic lesion target area was performed in accordance with the consensus guidelines for the delineation of target areas in NSCLC [18] as follows: the target area of the primary lesion in the lung was delineated in the lung window [1600, –600 Hounsfield units (HU)], and the mediastinal target area was delineated in the mediastinal window (400, 20 HU). The treatment regimen utilized involved-field irradiation without elective nodal irradiation (ENI). The target volumes were defined as follows: the gross tumor volume (GTV) was defined as the primary lesion in the lung or lymph nodes with a short diameter greater than 1 cm in the CT image; the clinical target volume (CTV) was defined as the GTV enlarged by margins of 8 mm (in cases of adenocarcinoma) or 6 mm (in cases of other pathologic types, such as squamous cell carcinoma and metastatic lymph nodes); and the planning target volume (PTV) was defined as the CTV enlarged by a margin of 10 mm to 15 mm based on the respiratory movement observed in the simulator. The GTV was determined by two radiation oncologists and one diagnostic imaging specialist. Two radiation oncologists outlined vital organs and body surface contours. Three to six coplanar fields were utilized for the conformal radiation. Dose-volume histograms (DVHs) were used to optimize the therapeutic plan. The treatment utilized 6 MV X-rays from a Siemens Primus Plus linear accelerator that was equipped with a 27-pair multi-leaf collimator (Topslane@_M, Shanghai Tuoneng Co., Shanghai, China).

The full course of accelerated hypofractionated radiotherapy for the thoracic lesions was conducted once daily at 3 Gy/fraction with 5 fractions per week, and this radiotherapy regimen was completed in 4–5 weeks.

Supraclavicular lymph node metastases received mixed irradiation with X-rays and electron rays with a conventional fraction of 2 Gy/fraction, once/day, 5 fractions/week, and the total dose was 60–70 Gy.

### Determination of the radiation dose

Regarding the radiation received by vital organs, the following limiting conditions were employed: the V20 (the percentage of healthy organ volume that receives 20 Gy radiation) for both lungs was no more than 30%; 0% of the esophagus was permitted to receive more than 70 Gy of radiation; a maximum of 10 cm of the esophagus was permitted to receive 60 Gy or more of radiation; 0% of the spinal cord was permitted to receive more than 40 Gy of radiation; and the V40 for the heart was no more than 40% [19]. Under the above constraints, the highest possible dose of radiation was applied, but the maximum dose did not exceed 75 Gy.

### Chemotherapy

Chemotherapy began on the first day of radiotherapy. NVB was administered by intravenous infusion at a dose of 25 mg/m^2^ on day 1 (d1) and day 8 (d8). CBP was administered at an area under the concentration-time curve (AUC) of 5 mg/ml on d8. This treatment was repeated every 28 days. At least 1 cycle of chemotherapy was performed concurrently with the radiotherapy. After the radiotherapy, the patients received a maximum of 4 cycles of consolidative chemotherapy, which utilized the same chemotherapy regimen that was employed during the concurrent chemoradiotherapy [19]. Anti-emetics, hepatoprotective drugs, and other treatments were also administered.

### Supportive care

To ensure the smooth implementation of the chemoradiotherapy regimen, patients whose absolute neutrophil count (ANC) decreased to less than 2.0 × 10^9^/L were administered granulocyte colony-stimulating factor (G-CSF) therapy until their ANCs increased to above normal levels. Patients with PLTs of less than 75 × 10^9^/L received interleukin-11 therapy until the PLTs increased to at least normal levels. If the patients developed esophageal symptoms, like dysphagia and odynophagia, 0.9% sodium chloride solution containing dexamethasone and lidocaine was immediately given to relieve the symptoms including pain and dysphagia, and to reduce the probability of the occurrence of severe esophagitis.Nutritional support via intravenous rehydration was also provided to patients as needed.

### Evaluation of adverse reactions

The Common Terminology Criteria for Adverse Events (CTCAE), version 3.0, which was issued by the National Cancer Institute/National Institutes of Health (NCI/NIH), was used as the standard for the evaluation of treatment toxicity. Weekly assessments of toxicity were conducted during the concurrent chemoradiotherapy treatment. Adverse events that occurred within 90 days from the beginning of radiotherapy were classified as acute toxicity, and those that occurred more than 90 days after the beginning of radiotherapy were classified as late toxicity.

### Evaluation of the short-term efficacy of tumor treatment

The evaluation of the short-term treatment efficacy was based on the thoracic-abdominal spiral CT examination results at 4 weeks after the completion of the radiotherapy. The Response Evaluation Criteria in Solid Tumors, version 1.1, (RECIST 1.1) standard was utilized for these assessments [20].

### Dose attenuation

Dose attenuation was implemented based on the most serious adverse events that occurred at any point after the start of the treatment.

Because we were conducting a feasibility study of high dose radiotherapy, no reductions in radiation dose were permitted. However, if a toxicity of grade III or higher occurred (with the exception of grade III nausea, vomiting, or weight loss), the radiotherapy was postponed until the toxicity had resolved. In contrast, if adverse events unrelated to radiotherapy occurred, such as peripheral neuropathy, the radiotherapy was continued, but the chemotherapy was suspended. The chemotherapy was resumed after these adverse events had dissipated.

The chemotherapy dose-adjustment procedures were as described below. Both radiotherapy and chemotherapy were suspended until the toxicity had resolved in the case of grade III/IV thrombocytopenia, grade III/IV anemia, grade IV neutropenia, or grade III/IV non-hematologic toxicities (except for grade III nausea, vomiting, or weight loss). If the toxicity had not resolved within 2 weeks, the patient was then excluded from the study. The NVB and CBP doses of the next chemotherapy cycle were reduced by 25%, and the patient received prophylactic G-CSF treatment. In the case of grade III neutropenia or grade II thrombocytopenia alone, chemotherapy was stopped, but the radiotherapy was continued. The NVB and CBP doses of the next chemotherapy cycle remained unchanged, and the patient received prophylactic G-CSF treatment.

### Follow-up and statistics

A follow-up was conducted every 3 months for the first 2 years after the completion of the radiotherapy and every 6 months thereafter. All of the statistical analyses were performed using the SPSS 19.0 biostatistical software package. The survival data were evaluated with the Kaplan-Meier method. The survival time was measured from the initiation of the concurrent chemoradiotherapy until death due to any cause or the subsequent follow-up event. Only the first treatment failure was taken into account. PFS was defined as survival without local recurrence or distant metastases.

## Results

### Patient characteristics

From June 2010 to August 2012, 27 patients with pathologically or cytologically confirmed NSCLC were enrolled in this study. Twenty cases had previously untreated NSCLC, and 7 cases had recurrent NSCLC. Of these, 26 patients received toxicity and efficacy evaluations (1 patient withdrew from the study due to personal reasons, rather than treatment toxicity). The clinical data for these patients are provided in Table 2. The median age was 68 years, and the median KPS score was 80. The cases included 9 cases of squamous cell carcinoma, 11 cases of adenocarcinoma, 2 cases of large cell carcinoma, 1 case of adenosquamous carcinoma, 1 case of sarcoma, and 2 cases of undifferentiated carcinoma. The 19 patients with previously untreated NSCLC included 6 cases of stage IIIA and 13 cases of stage IIIB (including 6 cases of supraclavicular lymph node metastasis).

**Table 2:** Patient Characteristics

**Characteristic**	**No. of patients**		**Percentage of**
	**N = 26**		**Patients (%)**
Gender			
Male	17		65.4
Female	9		34.6
Age			
Median		68	
Range		46-75	
Karnofsky performance status			
Median		80	
Range		70-90	
Histology			
Squamous cell carcinoma	9		34.6
Adenocarcinoma	11		42.3
large cell carcinoma	2		7.7
Adenosquamous carcinoma	1		3.8
sarcoma	1		3.8
Undifferentiated carcinoma	2		7.7
Stage			
IIIA	6		23.1
IIIB	13		50.0
Recurrence post-surgery post-surgery	7		26.9
GTV			
Mean		98.1 cm3	
Range		30.2-238.7 cm3	
PTV			
Mean		248.4 cm3	
Range		132.6-396.5 cm3	

### Compliance

Among the 20 patients with previously untreated NSCLC, 1 patient refused to continue treatment due to personal reasons, rather than treatment toxicity. This patient received only 15 Gy of radiotherapy and one cycle of NVB chemotherapy before withdrawing and could not be evaluated. However, 26 patients completed the radiotherapy with dose levels of 60 Gy-75 Gy. The 60 Gy, 63 Gy, 69 Gy, and 75 Gy groups included 12, 3, 6, and 5 participants, respectively, as presented in Table 3. Among the 26 patients who completed at least one cycle of concurrent chemotherapy, all received consolidative chemotherapy with a median of 4 chemotherapy cycles (with a range of 1 to 4 cycles).

**Table 3:** Patients in different groups of radiation dose

**3D-CRT hypo (Gy)**	**Cases**
60	12
63	3
69	6
75	5

### Toxicity

Table 4 contains the acute and late toxicities. No patients within the entire group had treatment-related death (Grade V). The common radiation-related toxicities included radiation esophagitis and radiation pneumonitis, but most of these toxicities were mild to moderate and could be easily treated in clinical practice. Except for 3 cases, all of the patients had esophagitis, and the total incidence was 88.5%. There were 4 patients with grade III esophagitis; 3 out of the 4 patients received a radiotherapy dose of 75 Gy, and the other patient received 69 Gy. Among the 3 patients in the 75 Gy group, the esophagus of 2 patients received a radiation dose as high as 75 Gy, which was above the pre-set constraints (less than or equal to 70 Gy). These 2 patients developed grade III late toxicities, and the esophageal stricture affected eating; therefore, esophageal dilatation was required. Late esophageal toxicity was generally mild and occurred in only 5 cases (19.2%). Except for the 2 cases who received the highest radiation dose of 75 Gy, the rest developed grade I/II conditions. Severe acute radiation pneumonitis was rare, with only 2 cases of grade III (7.7%). No grade III and above late pulmonary fibrosis occurred.

**Table 4:** Acute and late toxicities

**Item**	**Grade I**		**Grade II**		**Grade III**		**Grade IV**		**Total**	
	**Case**	**%**	**Case**	**%**	**Case**	**%**	**Case**	**%**	**Case**	**%**
**Acute**										
Radiation pneumonia	5	19.2	4	15.4	2	7.7	0	0	11	42.3
Cough	6	23.1	3	11.5	2	7.7	0	0	11	42.3
Radiation oesophagitis	10	38.5	9	34.6	4	15.4	0	0	23	88.5
Radiation dermatitis	3	11.5	2	7.7	0	0	0	0	5	19.2
Nausea	8	30.8	10	38.5	6	23.1	0	0	24	92.3
Vomiting	6	23.1	3	11.5	1	3.8	0	0	11	42.3
Anorexia	12	46.2	10	38.5	3	11.5	0	0	25	96.2
Fatigue	10	38.5	8	30.8	2	7.7	0	0	20	76.9
Neutropenia	4	15.4	14	53.8	5	19.2	3	11.5	26	100
Thrombocytopenia	8	30.8	4	15.4	1	3.8	0	0	13	50
Anemia	6	23.1	3	11.5	0	0	0	0	9	34.6
ALT	4	15.4	1	3.8	0	0	0	0	5	19.2
AST	3	11.5	0	0	0	0	0	0	3	11.5
Cr	1	3.8	0	0	0	0	0	0	1	3.8
BIL	2	7.7	0	0	0	0	0	0	2	7.7
**Late**										
Lung	12	46.2	4	15.4	0	0	0	0	16	61.5
Esophagus	1	3.8	2	7.7	2	7.7	0	0	5	19.2
Skin	1	3.8	0	0	0	0	0	0	1	3.8

*ALT* alanine aminotransferase, *AST* aspartate aminotransferase.

*Cr* serum creatinine, *BIL* bilirubin.

All (100%) of the patients exhibited neutropenia, and 30.8% exhibited grade III/IV neutropenia. In contrast, grade III/IV thrombocytopenia and anemia were rare. Nausea, fatigue, and loss of appetite were observed in most of the patients. However, these effects were mild to moderate and were successfully alleviated through the administration of appropriate antiemetics and intravenous rehydration without affecting the implementation of chemoradiotherapy. Liver and kidney toxicity was rare.

### Short-term treatment efficacy

Evaluations of the short-term treatment efficacy for the 26 cases were as follows: complete response (CR) 26.9% (7/26), partial response (PR) 53.8% (14/26), stable disease (SD) 19.2% (5/26), and no progressive disease (PD). The total response rate (RR) was 80.8% (21/26).

### Survival

**Figure 1:**
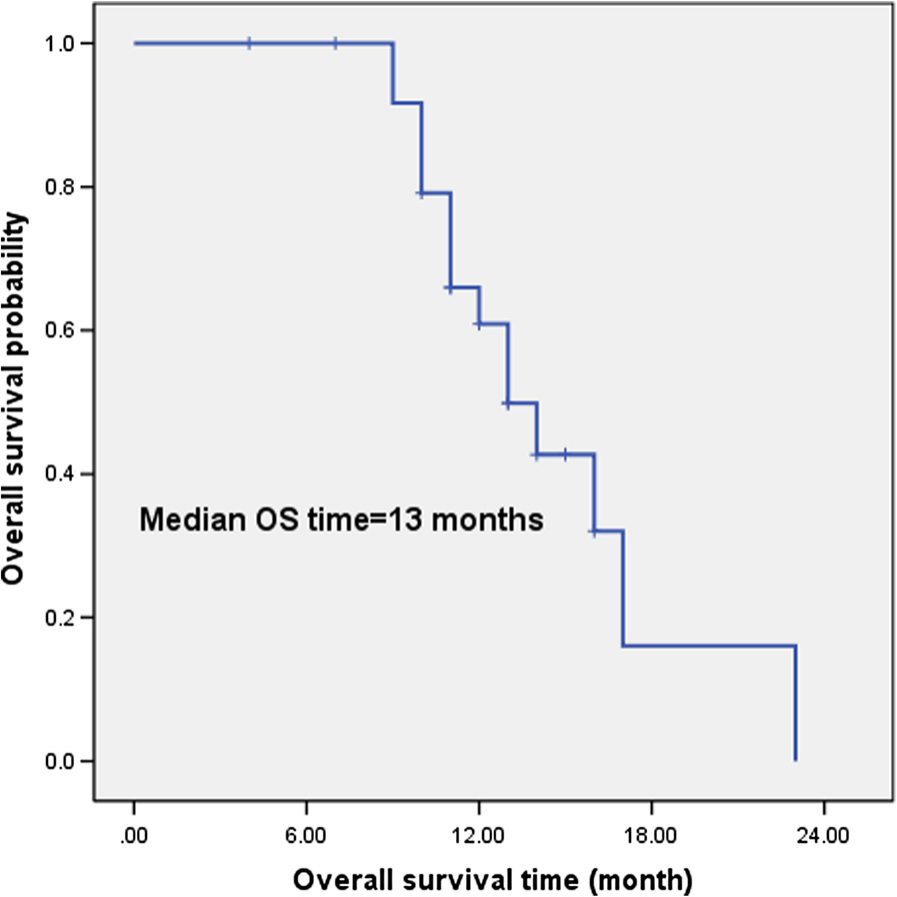
The median PFS time was 10 months, and 1- year PFS rate was 37.0%.

**Figure 2:**
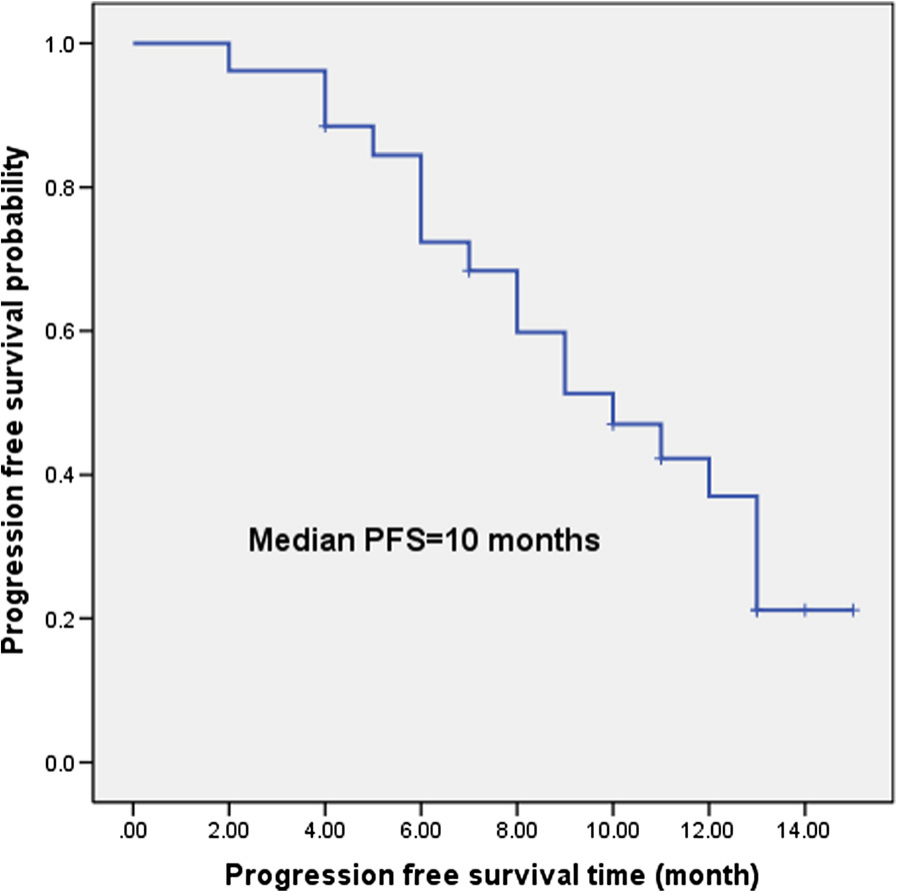
The median OS time was 13 months, and 1- year OS rate was 60.9%.

Although this study is a feasibility study, we nonetheless determined preliminary survival results. The median follow-up period was 11.5 months (4 months-23 months), the median PFS was 10 months, and 37.0% of the study participants achieved a 1-year PFS (Figure 1). The median OS was 13 months, and 60.9% of the study participants achieved 1-year OS (Figure 2). Eleven cases survived, and 15 cases died. The reasons for treatment failure were as follows: 3 cases had residual cancer or a recurrence within the radiation field; 2 cases had a recurrence within the radiation field and distant metastasis; and 13 cases had distant metastases ( 3 cases of liver metastasis, 3 cases of bone metastasis, 2 cases of brain metastasis, 1 case of adrenal metastasis, and 4 cases of multiple metastases). The treatment failure was mainly due to distant metastases, which was up to 72.2% (13/18).

## Discussion

NSCLC is a type of rapidly proliferating tumor. Studies have demonstrated that the median potential cell doubling time is 7 days [21], and accelerated proliferation occurs in the 3rd week-4th week of radiotherapy [22], which is one of the important reasons for NSCLC radiotherapy failure. If sufficient radiation is applied within a short period of time, this approach might overcome the accelerated repopulation. Accelerated hypofractionated radiotherapy can shorten the total treatment time, apply a high-dose of radiation in a short period of time, improve the biological effective dose (BED), and play an important role in the treatment of NSCLC [21]. However, due to the concerns of severe late toxicity, accelerated hypofractionated radiotherapy is not yet widely applied for the treatment of NSCLC. With improved three-dimensional conformal and intensity-modulated radiotherapy technologies, it is possible to accurately irradiate the target area while significantly reducing the radiation dose received by the surrounding normal tissues and organs [9]. Therefore, it is necessary to re-evaluate the tolerance for accelerated hypofractionated radiotherapy.

Fletcher [23] demonstrated that a radiotherapy dose of 80 Gy-100 Gy was required to cure lung cancer. Dose-escalation studies of three-dimensional conformal radiotherapy showed that in the 63 Gy-103 Gy range, a high radiation dose increased local control of the tumor and OS [24]. The clinical practice of stereotactic body irradiation for NSCLC yielded the same results: the survival of patients who received radiotherapy at a BED of at least 100 Gy was significantly better than those who received a BED of less than 100 Gy [25]. The dose of radiation can significantly affect the NSCLC treatment result.

Thirion et al. [26] conducted a study of high-dose accelerated hypofractionation at 3 Gy/fraction in which a 72 Gy radiation dose was delivered in 24 fractions over 5 weeks. The regimen was tolerated. One of the study participants experienced grade III radiation pneumonitis, 2 experienced grade III radiation esophagitis, and no grade IV or higher adverse events occurred among the entire group of participants. Xie et al. [16] performed a dose-escalation trial of accelerated hypofractionated radiotherapy at 3 Gy/fraction with cases from the Chinese population and found that patients tolerated the radiotherapy treatment at doses of up to 75 Gy at V20 levels no greater than 20% or at doses of up to 69 Gy at V20 levels between 20% and 30%.

It has been confirmed in the conventional fractionated radiotherapy of NSCLC that radiotherapy combined with chemotherapy is superior to radiotherapy alone. In vitro studies suggest that the combination of radiotherapy and chemotherapy can significantly increase the biological effects of hypofractionated radiotherapy [27]. Therefore, in theory, hypofractionated radiotherapy combined with chemotherapy should also be superior to hypofractionated radiotherapy alone. The Fudan University Cancer Center reported the results of a Phase II trial of hypofractionated radiotherapy with sequential chemotherapy. The first half of radiotherapy utilized a radiation dose of 50 Gy with 2.5 Gy fractions, and the dose was subsequently increased to 3 Gy/fraction to a total radiation dose of 65 Gy to 68 Gy, although ENI was not performed. All of the study participants received 2 cycles of induction chemotherapy with sufficient doses of NVB and cisplatin, and good treatment efficacy was observed. The median PFS, the median OS, and the 3-year OS rate were 10 months, 19.0 months, and 32.1%, respectively, with acceptable treatment toxicities [28].

Among the limited number of studies focused on hypofractionated radiotherapy with concurrent chemotherapy, each study utilized different fractionation and chemotherapy regimens [9-13]. The European studies used 2.75 Gy/fraction [9-12], while the South Korean study [13] applied 2.4 Gy/fraction [the simultaneous modulated accelerated radiation therapy (SMART) boost technology]. It is noteworthy that all three studies suggested that the toxicity could be tolerated and that hypofractionation did not seem to significantly increase the late toxicity, which is consistent with our conclusion. The study with the largest sample size was the European Organization for Research and Treatment of Cancer (EORTC) phase III trial [10], which had a total of 158 enrolled patients. Because the regimen for the concurrent group utilized concurrent daily low-dose cisplatin treatment, the hematologic toxicity of the concurrent group was significantly lower than that of the sequential group (provided the dose of chemotherapy was sufficiently high). Despite the application of ENI, the rate of grade III acute esophagitis in the EORTC study was similar to our study, 14% vs. 15.4%. In contrast, the rate of late esophageal toxicity in the EORTC study was lower than in our study, and the rates in the EORTC study and our results were 4% and 7.7%, respectively. The EORTC study had 2.75 Gy fractions and a relatively high total dose of 66 Gy, and in addition, that study utilized ENI. The mild toxicity observed in the EORTC study results might be related to the low doses of concurrent chemotherapy. The EORTC study indicated that the survival of the concurrent chemoradiotherapy group was not superior to that of the sequential group. This result might be due to the low-dose single-agent chemotherapy because some studies have suggested that the intensity of the chemotherapy dose is critical for concurrent chemoradiotherapy [29].

In our study, a relatively high radiation dose of 60 Gy-75 Gy was completed within 4 weeks-5 weeks. Five patients completed radiotherapy with doses up to 75 Gy. In addition, 2 patients received esophageal doses beyond our pre-determined constraints of esophageal dose, and the highest esophageal doses were both 75 Gy. These 2 patients both completed the full radiotherapy dose with persistent dysphagia. The grade III esophageal late toxicities that occurred 3 months later were esophageal stenoses, which necessitated esophageal dilatation. Until the last follow-up, the 2 patients were free of local progression. Among the remaining 3 patients whose esophageal doses did not exceed the constraints, 1 patient had grade III acute esophagitis that gradually dissipated after the end of the radiotherapy, and no late esophageal toxicity of grade III and above occurred. The radiotherapy at a dose of 75 Gy caused esophageal stenosis in 40% (2/5) of the patients within a short period of time, which should have been paid much more attention. Given that the radiotherapy at a dose of 75 Gy was performed in five patients along with a short follow-up, the safety of the radiotherapy at a dose of 75 Gy remains unknown. Prior to the publication of the phase I/II clinical trial results, the radiotherapy at a dose of 75 Gy should be avoided. All 5 patients had V20 below 30%, and there was 1 case of grade III acute radiation pneumonitis. No grade III or above late radiation-induced lung injury occurred among the entire group of participants. The main acute toxicities of all 26 patients were radiation esophagitis and pneumonia, which accounted for 88.5% (23/26) and 42.3% (11/26), respectively. Although the incidences of nausea, anorexia, and fatigue were high, these conditions did not affect the implementation of the chemoradiotherapy. Hematologic toxicity was common due to the sufficiently high doses of concurrent chemotherapy, with incidences of up to 100% for neutropenia, 50% for thrombocytopenia, and 34.6% for anemia. Because of our close monitoring and positive symptomatic and supportive treatment measures, there were no patients who had more than 7 days of interruption in the radiotherapy due to toxic effects. Due to the small sample size and short follow-up period, further longer term follow-up observations with larger sample size will be required in order to investigate the final safety and long-term toxicity for this treatment regimen. Based on the toxicity from the present study, an accelerated hypofractionated radiotherapy at a dose of 60 Gy or greater with concurrent chemotherapy with the NC scheme might be feasible.

The total incidence of the acute esophagitis in our study was as high as 88.5% (23/25), including 38.5% (10/25) grade I, 34.6% (9/25) II, and 15.4% (4/25) grade III esophagitis, which were comparable to those observed in several previous similar studies. A study conducted in South Korea reported a 81.6% (40/49) of total esophagitis and a 14.3% (7/49) incidence of the grade III/IV esophagitis following the involved-field radiotherapy [13]. In two studies of SOCCAR, the incidence of grade III esophagitis was 9.0% (6/67) and 13.3% (4/30), respectively with no grade IV or greater occurred [11,12]. Hypofractionated irradiation theoretically does not increase acute complications. The incidence of acute esophagitis in the current study was similar to what was observed in the conventional fractionated radiotherapy with concurrent chemotherapy. Furuse et al. [30] reported that the incidence of total acute esophagitis was 62.8% (98/156) with a 2.6% (4/156) incidence of grade III esophagitis. Furthermore, Zatloukal et al. detected a 18% incidence of grade III/IV esophagitis [31]. Our previous study reported a 78.4% (29/37) incidence of total esophagitis and a 13.5% (5/37) incidence of grade III acute esophagitis in the conventional 70-Gy fractionated radiotherapy with concurrent vinorelbine plus carboplatin chemotherapy [32]. A meta-analysis of seven clinical trials revealed a grade III/IV acute esophagitis in 18% of the 1077 patients [3].

However, there have been some studies reporting a significantly higher incidence for the acute radiation esophagitis (grade III or greater) as compared to what was observed in the current study. In the RTOG 94–10 study, the incidence of the grade III/IV radiation esophagitis was reported to be 23% (43/193) following the conventional 63 Gy fractionated radiotherapy [33]. In the NPC 95–01 study, the incidence of the grade III/IV radiation esophagitis was 32% following the conventional 66 Gy fractionated radiotherapy [34]. The high incidence of the radiation esophagitis in these two studies might have been associated with the preventive radiation of the lymph drainage area. In the RTOG 94–10 study, the radiotherapy at a dose of 45 Gy was given to the lymph drainage area [33]. In the NPC 95–01 study, a larger area received the radiation, including the ipsilateral hilum of the lung, mediastinum, and the homolateral supraclavicular fossa if the lesions occurred in the upper lobe of the lung. Furthermore, the paraesophageal and inferior pulmonary ligament nodal regions were exposed to radiation if the lesions occurred in the lower lobe of the lung [34]. This could significantly contribute to the radiation volume enhancement and an increased esophagus radiation dose, which could result in a high incidence of esophagitis.

In the current study, only the primary lesions with the mean gross tumor volume (GTV) of 98.1 (range, 30.2-238.7) cm3 and mean planning target volume (PTV) of 248.4 (range, 132.6-396.5) cm3 received the radiotherapy, resulting in a low radiation dose for the esophagus. The concurrent chemotherapy with the involved-field radiation technique at a dose of 74 Gy caused a relatively low incidence of severe acute radiation esophagitis. The RTOG 1107 study reported an 11.1% (1/9) incidence of esophagitis [4], while there was no occurrence of the grade III/IV esophagitis (0/6) in the NCCTGN 0028 study [5]. The incidence of grade III esophagitis was 16% (6/37) in the CALGB 30105 study, while no grade IV or greater esophagitis was observed [35]. Harada *et al.* reported the patients undergoing radiotherapy at a dose of 74 Gy did not develop the grade III/IV esophagitis (0/12) [36].

The EORTC study results indicated that the median survival time of the concurrent group was 16.5 months [10], similar to the median survival time of our study, which was of 13 months. The survival time in our study is significantly lower than the other two studies, which were 27.4 months [11] and 28.1 months [13], respectively. We believe that this result can be explained in the following ways: the South Korean study [13] and the United Kingdom study [11] were more stringent in selecting the enrolled patients, which might lead to a better prognosis. For example, the patient selection criteria required that the Eastern Cooperative Oncology Group (ECOG) score was 0–1 with the majority of patients having an ECOG score of 0. The patients were also required to have good lung function with a forced expiratory volume in 1 second (FEV1) of no less than 1 L or 0.75 L, etc. However, our study was a feasibility study. Regarding the performance status score, we only required that the KPS score was not less than 70, namely, the patients could take care of themselves most of the time. Furthermore, we had no explicit requirements for the FEV1, as long as the patients could receive chemoradiotherapy. The South Korean study [13] eliminated some poor prognostic factors, such as supraclavicular lymph node metastasis (which occurred in 6 patients in our study) and superior vena cava syndrome. In the United Kingdom study [11], squamous cell carcinoma accounted for 64% of all of the participants. Because squamous cell carcinoma is relatively less prone to distant metastasis, concurrent chemoradiotherapy might achieve better survival outcomes [37]. Of the patients who we enrolled in this study, 26.9% of them presented with a postoperative recurrence, which itself indicated a poor prognosis. Nevertheless, our concurrent chemoradiotherapy regimen still achieved a RR of 80.8%, the median and 1-year PFS values were 10 months and 37.0%, respectively, and the median and 1-year OS were 13 months and 60.9%, respectively. We believe that the current hypofractionated chemoradiotherapy regimen archives moderate efficacy and these survival results are acceptable.

The 3 hypofractionated concurrent chemoradiotherapy studies [10,11,13] noted above differed greatly from our study in the fractionation scheme and concurrent chemotherapy drugs. The doses per fraction used in the above 3 studies [10,11,13] and in our study were 2.4 Gy, 2.75 Gy, 2.75 Gy, and 3 Gy, respectively. The concurrent chemotherapy regimens included daily low-dose cisplatin [9,10], weekly paclitaxel and CBP [13], sufficient doses of NVB and cisplatin [11,12], and NVB and CBP (our study). It is difficult to compare directly the advantages and disadvantages of these chemotherapy regimens. From the existing literature, it is impossible to identify the optimal regimen of accelerated hypofractionated radiotherapy with concurrent chemotherapy or the maximum radiation dose to which the dose of accelerated hypofractionated concurrent chemoradiotherapy could be escalated.

In NSCLC radiotherapy and/or chemoradiotherapy, the lung is one of the most critical limiting organs. Studies focusing on the tolerated radiation dose of the lung are the most numerous and most mature. Because the lung is a parallel organ, its limiting dose is often measured by dose-volume parameters, such as V20 and V_eff_. The dose escalation of radiotherapy for the treatment of NSCLC often involves different bins of dose escalation based on different dosimetric of lung irradiation dose-volume. For patients with a smaller irradiation volume, the dose could be incremented to 103 Gy [24]. There are relatively fewer studies focusing specifically on the limiting dose of the esophagus. The conclusions of dosimetric parameters, such as the esophageal length of irradiation and radiation dose-volume, are not consistent [38-42]. Even for hyperfractionated radiotherapy alone, the esophagus is not considered to be a dose-limiting organ [43]. However, hypofractionated radiotherapy can theoretically increase late toxicity, and the esophagus must be considered as one of the dose-limiting organs. Currently, there are no unified limiting-dose parameters that can be applied to the esophagus. In the 2 European studies of hypofractionated concurrent chemoradiotherapy, a maximum of 12 cm of the esophagus in the PTV was permitted to receive radiation [10,11]. The South Korean study used the dose-volume parameters with V55 less than or equal to 30% [13]. Because the esophagus has the characteristics of both a parallel and a serial organ, we believe that the highest radiation dose received by the esophagus should also be considered. Based on the esophageal limiting dose in our conventional fractionated radiotherapy with concurrent chemotherapy, we chose the restriction conditions of a maximum irradiated length of 10 cm and a maximum dose of 70 Gy [19]. Under these constraints, the incidence of grade III and above acute esophagitis was only 8.3% (2/24), and there were no grade III and above late toxicities. In contrast, the 2 patients who received the highest esophageal irradiation dose of 75 Gy both had grade III esophagitis and developed a grade III late toxicity, which suggests that esophageal toxicity is also related to the highest dose of radiation. The various esophagitis evaluation criteria are not the same (RTOG/EORTC 1995 and CTC standards). In addition, the evaluation criteria for esophagitis, especially acute esophagitis, are quite subjective, and the grades of radiation esophagitis determined by different treatment centers might have significant differences. We thus found it difficult to determine which limiting dose of the esophagus was more reasonable and accurate [9-13]. Based on the above reasons, it is also difficult to determine the maximum tolerated dose in the absence of a unified esophageal limiting dose. Therefore, some studies have suggested that when conducting a dose escalation of radiotherapy for the treatment of NSCLC, the principle of individualization should be applied, and each case should be given a different highest dose so that each case would reach the maximum tolerated dose for normal tissue [43].

## Conclusion

In China a large part of patients were worried about the toxicity associated with the high-dose accelerated hypofractionated radiotherapy with concurrent chemotherapy that resulted in some difficulties in the recruitment. Therefore, a small-sample exploratory study was carried out. This study had three limitations, including: (1) Small sample size, with only 27 cases involved. (2) Short follow-up period, with the median follow-up period of only 11 months. (3) No strict inclusion/exclusion criteria for the subjects, which might have led to unsatisfactory survival outcomes. Therefore, a very definitive conclusion could be hardly acquired from the results. However, this prospective, exploratory study firstly evaluated the safety of the accelerated hypofractionated radiotherapy at a dose of >60 Gy with concurrent chemotherapy. The results suggested that: first, under certain constraints for normal tissue, high-dose hypofractionated three-dimensional conformal radiotherapy (60 Gy or greater at 3 Gy/fraction) with concurrent NVB and CBP chemotherapy might be feasible. Second, special attention should be paid to the esophageal toxicity caused by the high-dose hyopfractionated radiotherapy, particularly late toxicity. The safety of the hypofractionated radiotherapy at a dose of 75 Gy remains controversial. Therefore, the hypofractionated radiotherapy at a dose of 75 Gy should be avoided before the publication of the clinical trial results could verify its safety. Third, we recommend that an individualized dose-escalation study should be carried out by grouping patients based on the different risks of adverse events to obtain the maximum tolerated dose of accelerated hypofractionated concurrent chemoradiotherapy. The constraints of the lung and the esophagus should both be considered for radiotherapy dose escalation.

## Abbreviations

NSCLC: Non-small cell lung cancer; hypo: Hypofractionated radiation therapy; 3D-CRT: Three-dimensional conformal radiation therapy; NVB: Vinorelbine; CBP: Carboplatin; KPS: Karnofsky performance status; CR: Complete response; PR: Partial response; SD: Stable disease; PD: Progressive disease.

## Competing interests

The authors declare that they have no competing interests.

## Authors’ contributions

QL was the PI of this clinical trial, who designed the subject and helped to draft the manuscript. YL participated in the subject of design and draft the manuscript. FM participated in the design of the subject and carried out the clinical implementation of the study. XC participated in the design of the subject and carried out the clinical implementation of the study. XR carried out the clinical implementation of the study. BC guided the design of the subject and helped to draft the manuscript. NW carried out the clinical implementation of the study. JZ carried out the clinical implementation of the study. YP carried out the clinical implementation of the study. YK carried out the clinical implementation of the study. YC carried out the clinical implementation of the study. All authors read and approved the final manuscript.

## Authors’ information

QL, the corresponding author, is the Associate Professor of Department of Oncology, North China Petroleum Bureau General Hospital of Hebei Medical University, 8 Huizhan Avenue, Renqiu City, Hebei Province, and P.R. China. He is focusing on the chemoradiotherapy on the thoracic neoplasm. He has found difference tolerance between Asian patients and Western patients when they received concurrent chemoradiotherapy in lung cancer and esophageal carcinoma.
